# Recent Advances in the Immunoassays Based on Nanozymes

**DOI:** 10.3390/bios12121119

**Published:** 2022-12-02

**Authors:** Lu Zhou, Yifan Liu, Yang Lu, Peirong Zhou, Lianqin Lu, Han Lv, Xin Hai

**Affiliations:** College of Chemistry and Chemical Engineering, Research Center for Intelligent and Wearable Technology, Qingdao University, Qingdao 266071, China

**Keywords:** immunoassays, nanozymes, signal amplification, target detection

## Abstract

As a rapid and simple method for the detection of multiple targets, immunoassay has attracted extensive attention due to the merits of high specificity and sensitivity. Notably, enzyme-linked immunosorbent assay (ELISA) is a widely used immunoassay, which can provide high detection sensitivity since the enzyme labels can promote the generation of catalytically amplified readouts. However, the natural enzyme labels usually suffer from low stability, high cost, and difficult storage. Inspired by the advantages of superior and tunable catalytic activities, easy preparation, low cost, and high stability, nanozymes have arisen to replace the natural enzymes in immunoassay; they also possess equivalent sensitivity and selectivity, as well as robustness. Up to now, various kinds of nanozymes, including mimic peroxidase, oxidase, and phosphatase, have been incorporated to construct immunosensors. Herein, the development of immunoassays based on nanozymes with various types of detection signals are highlighted and discussed in detail. Furthermore, the challenges and perspectives of the design of novel nanozymes for widespread applications are discussed.

## 1. Introduction

**Scheme 1:**
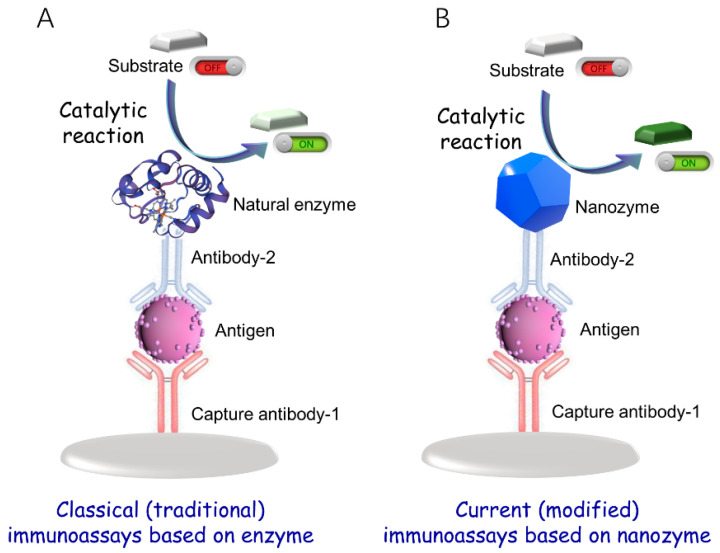
Schematic diagram of (**A**) classical (traditional) immunoassays based on natural enzyme and (**B**) current (modified) immunoassays based on nanozyme.

Immunoassay is a highly selective diagnostic method established by the specific immune reaction between antigens and antibodies. There are many kinds of immunoassays with various detection signals, such as colorimetric immunoassays, fluorescence immunoassay, chemiluminescent immunoassay, electrochemical immunoassay, electrochemiluminescence immunoassay, and so on. Typically, the detection is usually achieved by using various signal labels on the antibodies, such as radioactive isotopes [1], fluorophores [2], and enzymes [3]. Therefore, due to the high biocatalytic efficiency of enzymes, it has become a widely used label to construct enzyme-linked immunosorbent assays (ELISAs) [4]. The enzyme is either bound to an antibody or an antigen to catalyze the conversion of chromogenic substrates into visible colorimetric outputs, thus amplifying the signal to a great extent (Scheme 1A) [5]. Because of the merits of easy operation, high sensitivity and selectivity, automated high-throughput, and visual inspection, ELISA has become an attractive method in immunoassay, which has widespread applications, including clinical diagnosis, food safety, and environmental monitoring, whereas the natural enzymes labels usually suffer from some intrinsic drawbacks like low stability, high cost, and difficult storage [6]. Thus, the seeking of enzyme substitute in immunoassay is of vital significance.

With the development of nanotechnology and biotechnology, a new type of nanomaterials with intrinsic enzyme-like characteristics as “nanozymes” has been exploited in recent years [7]. Nanozymes not only show high catalytic activity with tunability but also eliminate the defect of natural enzymes, exhibiting the merits of easy preparation, low cost, and high stability [8]. Thus, nanozymes have become a charming substitute for natural enzymes. Up to now, a variety of nanozymes have been discovered, which can be classified into different categories. According to the structure and components, nanozymes can be classified into metallic nanozymes, such as ferroferric oxide (Fe_3_O_4_) nanoparticles [9,10], ceric oxide (CeO_2_) [11,12,13], and gold nanoparticles (AuNPs) [14,15]; nonmetallic nanozymes, like graphene quantum dots (GQDs) [16,17,18], carbon dots (CDs) [19], and graphite-carbon nitride (g-C_3_N_4_) [20]; and composite nanozymes. These nanozymes possess different catalytic mechanisms [21], serving as mimic enzymes, including peroxidase, oxidase, glucose oxidase, and so on, which can be designed as labels in constructing immunoassays to realize considerable detection sensitivity and selectivity [22].

By replacing natural enzymes with nanozymes, modified immunoassays based on nanozymes have emerged, which exhibit higher sensitivity and stability than traditional enzyme-immunoassays (Scheme 1B). So far, although a series of reviews have been published for the summarization of immunoassays with various detection signals, only a few focus on the employment of nanozymes. To attract the researchers interested in this charming field, a comprehensive summary and analysis of the advances of immunoassays based on nanozymes is urgently needed. In 2019, Niu et al. have published a review to summarize the nanozyme-based immunosensors and immunoassays. Inspired by this, herein, we present the recent progress in the construction of immunoassays based on nanozymes, especially reports from the last four years (2019–2022) [23]. Specifically, the classification of the immunoassays based on nanozymes goes according to the various signaling modes, including colorimetric, fluorescent, chemiluminescent, electrochemical, electrochemiluminescent, surface-enhanced Raman, photoelectrochemical signals, and other kinds of signals, as well as the combination of multimodal signals. In addition, methods for improving detection sensitivity based on nanozymes, including the use of double nanozymes, single-atom nanozymes, cascade catalytic amplification, and enrichment amplification, are also highlighted. Table 1 summarizes some typical immunoassays with diverse signals based on nanozymes for target analyte detection. Furthermore, the potential challenges and future trends in the advance of nanozyme-based immunoassays and their further applications are prospected.

## 2. Immunoassay Based on Nanozymes

### 2.1. Colorimetric Immunoassays Based on Nanozymes

The traditional immunoassay can be divided into enzyme-linked immunosorbent assay and immunochromatography assay. Because of the the characteristics of simplicity, high efficiency, and low cost, the colorimetric immunoassay has become one of the most commonly used immunoassays. With the features of easy preparation, low cost, and high stability, nanozymes have been widely used in immunoassays as good substitutes for natural enzymes. Generally speaking, a colorimetric immunoassay based on nanozymes is constructed by using the nanozymes coupling antibody to catalyze the color reaction directly or indirectly, and the change of color or signal intensity produced by the catalytic reaction is linear with the target concentration. In summary, colorimetric immunoassays based on nanozymes can be classified into two categories—spectrophotometry and immunochromatography.

#### 2.1.1. Plate-Based Colorimetric Immunoassays

##### Double Antibody Sandwich Colorimetric Immunoassays

**Figure 1:**
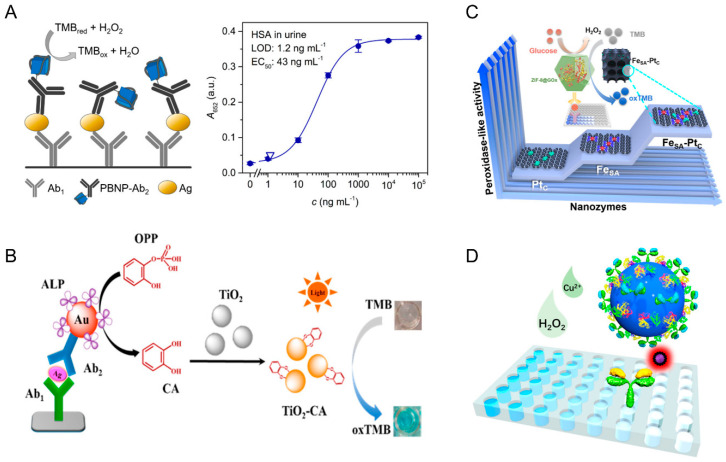
(**A**) Schematic diagram of PBNP-based sandwich NLISA [26]. Reprinted with permission from ref. [26]. Copyright 2018 American Chemical Society. (**B**) Scheme of mechanism of catalytic oxidation of TMB by photo-activated TiO_2_−CA for the quantitation of ALP [30]. Reprinted with permission from ref. [30]. Copyright 2015 American Chemical Society. (**C**) Scheme of the cascade reaction for the detection of PSA by FeSA−PtC nanozymes based ELISA [35]. Reprinted with permission from ref. [35]. Copyright 2021 American Chemical Society. (**D**) Scheme of sandwich-type TLISA for the detection of IL-6 by using TMB NPs [37]. Reprinted with permission from ref. [37]. Copyright 2019 American Chemical Society.

In classical colorimetric analysis, reaction color changes can be directly observed [24], but most of them are combined with photometric measurements [25,26,79,80]. Usually, a sandwich structure is formed in nanozyme-linked immunosorbent assay (NLISA) to measure some proteins, bacteria, and other targets with many antigen sites on their surfaces. Yan et al. constructed a typical colorimetric enzyme-linked immunosorbent assay (ELISA) by using amorphous RuTe_2_ (a-RuTe_2_) nanorods as peroxidase mimics, which could be labels to detect a prostate-specific antigen (PSA) [25]. This a-RuTe_2_ nanorods displayed ignorable oxidase-like activity that was beneficial to reduce the interference of O_2_ in a colorimetric assay. Farka and co-workers coupled Prussian blue nanoparticles (PBNPs) with peroxidase-like activity to antibodies to construct a nanozyme-linked immunosorbent assay (NLISA). [26]. Firstly, capture antibody (Ab1) was immobilized on the microtiter plate, which could specifically bind with antigen (Ag), such as the human serum albumin (HAS). Then a sandwich structure was formed upon the addition of antibody-modified PBNPs (PBNPs-Ab2), which could catalyze the oxidation of TMB in the presence of H_2_O_2_ to obtain colorimetric readout. Finally, HAS in urine was detected with a limit of detection of 1.2 ng·mL^−1^, confirming the applicability of PBNPs as a universal and robust alternative to enzyme tags (Figure 1A). In addition to the mimic peroxidase activity, light-responsive nanozymes have also been exploited to construct a photosensitive colorimetric immunoassay for carcinoembryonic antigen (CEA) detection by using Cu^2+^/Ag-AgI as labels [81]. Sometimes, nanozymes themselves also show ultraviolet–visible (UV–vis) absorption, generating a high background signal and a low signal-to-noise (S/N) ratio. Therefore, detecting the change in UV–vis absorption (ΔA) is necessary [27,28]. A colorimetric immunosensor was developed for the detection of tumor-associated anti-p53 autoantibodies (antip53aAbs), using protein G functionalized nanomagnet-silica nanoparticles decorated with Au@Pd (Fe_3_O_4_@SiO_2_-NH_2_-Au@Pd_0.30_NPs-protG) as the nano-biological probe. Fe_3_O_4_@SiO_2_-NH_2_-Au@Pd_0.30_NPs-protG not only had high affinity for the captured anti-P53AAbs but also exhibited a great catalytic performance for the oxidation of 3,3,5, 5-tetramethylbenzidine (TMB) [27]. 

To make the detection system more portable, the spectrophotometer can be replaced with a smartphone to record the signal [29,82,83]. Zhang and co-workers prepared a new type of laccase mimics (LM) with strong catalytic activity, good affinity with substrates, and high stability using the hydrothermal method, which could catalyze the oxidative coupling reaction of 2,4-dichlorophenol (2,4-DP) and 4-aminopyrine (4-AP) to produce obvious red products [29]. Based on this, LM nanozymes were used as a potent substitute for natural enzymes to build NLISA of alpha-lactalbumin (allergenic protein). Later, NLISA can be integrated with the smartphone to create a high-throughput and portable detection method. This work not only makes the types of nanozymes more diverse but also makes the nanozymes have a wider application prospect in the field of biosensing. 

In order to further improve the detection sensitivity, the signal amplification strategy is an alternative. One way to achieve an amplified signal is to use a dual-enzyme with improved enzymatic activity [32,33]. Liu et. al. established an immunosensor for the visual, sensitive, and selective detection of microcystin-lr (MC-LR) using a coupling nanozyme with G-quadruplex/hemin DNAzyme to form a double-integrated Cu(OH)_2_ nanozyme with excellent peroxidase activity [33]. The finely regulated Cu(OH)_2_ nanocages could be used as labels to capture the secondary antibody of the immunoreaction and the proliferating DNA primer, which was amplified by hybridization chain reaction, thereby producing a large number of DNAzymes (G-quadruplex/Hemin) on the surface of Cu(OH)_2_ nanocages. To further enhance the catalytic activity, a nanozyme nest composed of an iron-based metal–organic framework (Fe-MOF) and graphene oxide (GO) with a rich 2D catalytic interface structure was synthesized, to construct ultrasensitive immunosensors for the quantification of a woodsmoke biomarker [32]. The synergistic effect of a large surface area and abundant active sites of graphene and Fe-MOF made the nanozyme nests exhibit amplified peroxidase-like catalytic ability. 

Likewise, the self-linkable dual-nanozyme, Prussian blue (PB)-incorporated magnetic graphene oxide (PMGO), showed excellent peroxidase catalytic activity, for the construction of a colorimetric immunosensor [84]. Although Prussian blue (PB) nanoparticles (NPs) possess peroxidase-like activity, it is hard to use them as signal labels in sandwich-type colorimetric immunoassays. To solve this issue, Li and co-workers developed a highly efficient PB-based colorimetric immunoassay that used polydopamine (PDA)-Fe (III) NPs as signal labels to detect biomarkers [39]. The PDA-Fe (III) NPs achieved the high loading of Fe (III) ions through chelating interactions. Fe (III) ions could be rapidly released in solution via the pH-induced decomposition of PDA to generate PB NPs in situ. Due to the high peroxidase-like activity of the PB NPs and the excellent catalysis triggered by colorimetric reaction, the detection sensitivity of prostate-specific antigen (PSA) was significantly improved.

Another method for signal amplification is to construct an enzymatic cascade reaction system [30,31]. For instance, an enzyme cascade amplification immunoassay (ECAIA) was exploited for the detection of alpha-fetoprotein (AFP) based on nanobody-alkaline phosphatase fusion (Nb-ALP) and MnO_2_ nanoflakes with oxidase-like activity [31]. Besides the common enzyme cascade reaction, Jin and co-workers proposed a new concept, in which catechol (CA) generated in the in situ biocatalytic process of ALP and then complexed with TiO_2_ to form phototriggered nanozyme-CA coordinated TiO_2_ (TiO_2_-CA), which exhibited oxidase-like activity under visible light stimulation (λ 400 nm) [30]. Thus, a colorimetric biosensor for detecting ALP activity and its inhibitor of 2,4-dichlorophenoxyacetic acid (2,4-Da) was designed. This method has high sensitivity and can quantitatively detect ALP activity in the linear range of 0.01 to 150 U/L with a detection limit of 0.002 U/L (Figure 1B). 

In recent years, single-atom catalysts have been found, with boosted enzyme-like activity [35,85]. The catalytic activity of an enzyme is closely related to the coordination environment; thus, rationally designing the coordination structure of the active site at the atomic scale provides a new idea for the development of high-performance enzyme-like catalysts. Inspired by this, Zhu’s groups investigated structure–activity relationships through pentacoordinated and tetracoordinated Fe-N-C single-atom catalysts (named NG-Heme and G-Heme, respectively) [85]. The result indicated that the push effect of the additional axial ligands significantly enhanced the peroxidase-like catalytic activity of NG-Heme. Encouraged by the outstanding catalytic activity, a NG-Heme-linked immunosorbent assay was established for the colorimetric detection of carcinoembryonic antigen (CEA). Meanwhile, this group also synthesized highly active peroxidase-like nanozymes by loading Pt clusters on Fe single atoms (FeSA-PtC) [35]. Due to the synergistic effect between Fe single atoms and Pt clusters, the FeSA-PtC nanozyme-like peroxidase activity is 4.5-fold and 7-fold higher than that of FeSA and PtC nanozymes, respectively. To combine FeSA PtC nanozymes with glucose oxidase, a cascade signal amplification strategy for prostate-specific antigen was proposed, which had good sensitivity and high selectivity with a detection limit of 1.8 pg/mL (Figure 1C).

In some cases, the enrichment of the target analytes can also boost the detection sensitivity [36,86]. Magnetic separation is a widely used method for biomarker enrichment. A magnetic nanozyme-linked immunosorbent assay (MagLISA) was presented for the ultrasensitive detection of influenza virus A [36]. The MagLISA was integrated with three functional components, including (i) the magnetic nanobeads for biomarker enrichment, (ii) gold nanozymes for peroxidase-like artificial catalyst, and (iii) anti-hemagglutinin monoclonal antibody for a specific recognizer. Such a sensing platform with sample separation, enrichment, ultrasensitive readout, and anti-interference capability makes the early diagnosis of influenza virus feasible. 

In addition to the above methods, signal amplification can be achieved by direct or indirect introduction and the specific generation of certain substances, such as 3,3′,5,5′-tetramethylbenzidine (TMB) [37,38,87]. For instance, TMB could not only serve as the chromogenic substrate in an enzymatic catalysis system but also as a reducing agent to synthesize gold nanoparticles (Au NPs), which was generated from a viral target-specific antibody-gold ion mixture with a blue color. Then, the generated Au NPs served as a peroxidase-like nanozyme to further amplify the blue color by catalyzing the TMB/H_2_O_2_ system. The assembly of the allochroic molecules (like TMB) is a choice to obtain signal tags for signal amplification. Jiao and co-workers prepared an all-inclusive metachromatic TMB NPs based on the self-assembly strategy for developing improved ELISA (named TLISA) [37]. In the process of biomarker detection, plentiful TMB molecules enriched by the self-assembly of TMB molecules were oxidized by Cu^2+^/H_2_O_2_, which could produce an obvious chromogenic reaction. The sensitivity of TLISA to inflammatory biomarkers interleukin-6 was 11.82 times higher than that of conventional ELISA, which laid the foundation for the development of ultrasensitive colorimetric immunoassay (Figure 1D). 

It is reported that silver ions can regulate the catalytic activities of some nanozymes (such as AgNPs) [34,40,88,89]. Khoris et. al. pre-deposited silver on the gold nanozyme to form the silver shell and then used hydrogen peroxide to decompose the silver shell to generate Ag^+^ [88]. Ag^+^ can significantly improve the oxidation ability of TMB to achieve signal amplification. Guo and co-workers utilized silver ions to activate the mimic peroxidase activity of cetyltrimethylammonium bromide (CTAB)-coated gold nanoparticles (AuNPs@CTAB) and used AuNPs@CTAB with Ag^+^-triggered catalytic activity as the signal sensor to construct a colorimetric immunoassay for the detection of human chorionic gonadotropin (HCG) [40]. Abundant Ag^+^ were released through the corrosion of silver nanoparticles by hydrogen peroxide. Furthermore, this work also synthesized three AuNPs@CTABs with similar sizes but different shapes, including nanospheres (AuNSs@CTAB), nanorods (AuNRs@CTAB), and nanocubes (AuNCs@CTAB). Among them, AuNSs@CTAB showed the best catalytic performance, which can make the detection limit reach 7.8 mIU/mL. 

##### Competitive Colorimetric Immunoassay

Competition methods are often used to detect small antigens that are difficult to couple with two antibodies simultaneously. Nanozymes show excellent stability and catalytic activity, which are promising substitutes for natural enzymes in immuno-sensing. Chen and co-workers designed a competitively sensitive biological barcode immunoassay method based on peroxidase-mimicking bimetallic nanozyme (Au@Pt) catalysis and used it for the detection of the pesticide parathion [41]. First, single-stranded thiol oligonucleotides (ssDNAs) and monoclonal antibodies (mAbs) were modified on gold nanoparticle (AuNP) probes. Au@Pt probes were formed by functionalizing with the complementary thiolated single-chain thiol oligonucleotides (ssDNA). Then Au@Pt probes reacted with AuNP probes through complementary base pairing. Afterwards, parathion competed with MNP probes (magnetic nanoparticles (MNPs)-coated ovalbumin (OVA) parathion haptens) to bind monoclonal antibodies to the AuNP probes. Finally, the Au@Pt nanozymes were liberated from the complex to catalyze the oxidation of TMB. However, the traditional competitive colorimetric method usually exhibits a signal-off mode, which cannot produce distinctive color changes. To avoid this problem, Ma et. al. used silver ions to modulate the activity of peroxidase-mimicking nanozyme (polyvinylpyrrolidone (PVP)-capped Pt nanocubes (PVP-PtNC)) and converted the traditional signal-off model to a signal-on model, which was beneficial in improving the sensitivity of drug detection [42]. 

Up to now, most of the nanozyme-based colorimetric immunoassay utilized mimic peroxidases, whereas oxidase-like nanozymes are more desirable, owing to the omission of H_2_O_2_ [43,90,91]. Zhu and co-workers synthesized well-dispersed Co(OH)_2_ nanocages through a template-assisted strategy, which could oxidize TMB in the absence of H_2_O_2_, for the development of a simple and sensitive colorimetric immunoassay to detect ochratoxin A (OTA) [43]. By comparing the activities of Mn_2_O_3_, Mn_3_O_4_, and MnO_2_, it was found that Mn_2_O_3_ had the highest oxidase activity [90]. In view of the weak physical adsorption ability of Mn_2_O_3_ to antibodies, a covalent coupling method was established. Coating Mn_2_O_3_ with amine-containing silane and then coupling the antibody with glutaraldehyde, a one-step indirect competitive ELISA (icELISA) was established to detect isocarbophos, and the obtained IC50 was 261.7 ng/mL. 

#### 2.1.2. Immunochromatography Based on Nanozymes

Traditional immunochromatographic assay (ICA) often make use of colloidal gold as markers to achieve a color reaction. With the continuous development of nano-labeled materials, nanozymes can work as natural enzymes to catalyze substrates with a color change, thereby enhancing the detection signal of ICA and greatly improving the detection sensitivity. Therefore, nanozymes are widely used as markers in ICA. The detection principle of ICA is mainly divided into two categories, the double antibody sandwich method and competition method. The double antibody sandwich method is appropriate for the detection of polyvalent antigens with at least two antigenic determinants, while the competition method is suitable for the detection of small-molecule antigens that cannot bind to both antibodies. 

##### Double Antibody Sandwich Method

**Figure 2:**
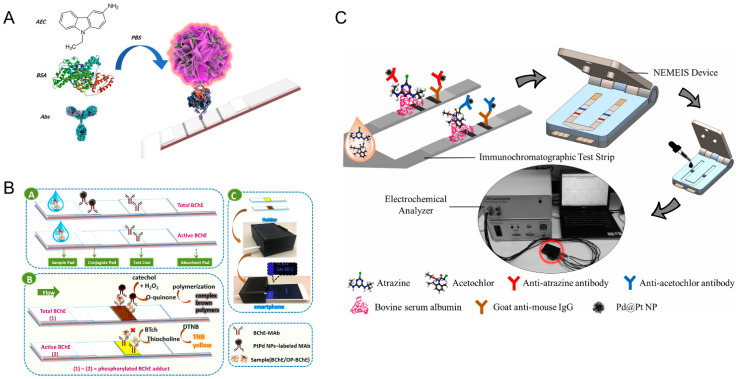
(**A**) Scheme of the detection principle of cardiac biomarkers based on SANs-lateral flow immunoassay [44]. Reprinted with permission from ref. [44]. Copyright 2021 American Chemical Society. (**B**) Schematic diagram of mechanism of the simultaneously immunodetection of the BChE activity and total amount of BChE [51]. Reprinted with permission from ref. [51]. Copyright 2018 American Chemical Society. (**C**) Scheme of nanomaterial-enhanced 3D-printed sensor platform for simultaneous detection of two herbicides [53]. Reprinted with permission from ref. [53]. Copyright 2021 Elsevier.

In the typical nanozyme-based immunochromatographic assay (ICA) [44,45], nanomaterials with enzyme-like activity as ICA probes can reduce the interference of the matrix in the sample and improve the detection sensitivity and specificity. A polydopamine (PDA)-mediated magnetic bimetallic nanozyme (Fe_3_O_4_@PDA@Pd/Pt) with peroxidase-like activity was used as the ICA probe [45]. The magnetic property of Fe_3_O_4_@PDA@Pd/Pt could effectively enrich targets, thereby reducing the interference of the matrix in the sample to the detection. The nanozyme-based ICA can also be applied to the detection of multiple analytes. Song and co-workers designed self-assembled allochroic nanoparticles (SANs) with peroxidase-like activity assemblies [44]. SANs assemblies not only reduced the number of liquid handling steps involved in sample analysis, but also could be used to detect troponin I and myoglobin simultaneously. There were three color bands in this ICA, and SANs encapsulated chromogenic molecules (3,3,5,5—tetramethylbenzidine (TMB) and 3-amino-9-ethylcarbazole (AEC) molecules) in individual nanoparticles (NPs) through hydrophobic interactions. After the oxidation reaction with Cu^2+^/H_2_O_2_, the NPs generated color change and were enriched in test line (T lines) containing different trapping agents, resulting in two bands of different colors (Figure 2A).

Unlike typical ICA that only relies on the raw signal intensity on the T line as the quantitative indicator, Wang and co-workers used a functional nanozyme, mannose-modified Prussian blue (man-PB), as a recognition agent and signal indicator, thereby establishing a multi-readout and label-free lateral flow immunoassays (LFIAs) to rapidly detect *Escherichia coli* O157:H7 (*E. coli* O157:H7) [46]. The man-PB nanoparticles with peroxidase-like catalytic activity could not only maintain the original bright blue color (used for qualitative and quantitative detection) but also obtained colorimetric signals through catalysis (used for quantitative detection). This multi-readout strip sensor has the potential to enable a variety of detections of real samples in complex areas.

Detection methods with higher sensitivity and lower detection limits have been the pursuit of researchers. Nanozymes can serve as excellent optical reporters. Cheng et. al. synthesized mesoporous core shell palladium@platinum (Pd@Pt) nanoparticles with peroxidase-like activity, which were used as the signal amplifier in dual LFIAs for the simultaneous detection of *Salmonella Enteritidis* and *E. coli* O157:H7 [47]. Bradbury and co-workers first proposed an aqueous two-phase system (ATPS)-automated concentration and enhancement of the lateral-flow immunoassay (ACE-LFA) [48]. ACE-LFA could not only control the time of reagent delivery by changing the initial composition of the ATPs but also innovatively realized the automated biomarker concentration, capture, and nanozyme signal enhancement in a single paper-based device. Compared with traditional LFA, ACE-LFA had a 30-fold improvement in the detection limit in the detection of *E. coli*, maintaining the original simple operation steps. Furthermore, a cascade-signal amplification strategy is known to enable sensitive detection. a two-step cascade signal amplification strategy combining in situ gold growth and nanozyme-catalyzed deposition was proposed, for the sensing of *E. coli* with a detection limit of 1.25 × 10 CFU/mL, which was nearly 400-fold lower than colloidal gold immunochromatography assay (AuNP-ICA) (5 × 10^3^ CFU/mL) [49]. For simultaneous visual detection and quantification, Zhang and co-workers designed a continuous cascade nanozyme sensor to detect viable *Enterobacter sakazakii* (ES), based on propidium monoazide (PMA), loop-mediated isothermal amplification (LAMP), and a nanozyme (Fe_3_O_4_ magnetic nanoparticles) strip [50]. PMA were utilized for distinguishing DNA in viable ES and dead ES; LAMP process could amplify DNA and form dual-labeled products, and, finally, magnetic nanoparticles of Fe_3_O_4_ were used as nanozyme probes to realize the visual detection of ES. 

Moreover, the ubiquity and versatility of smartphones can be used as a terminal device to achieve portable and easy-to-operate immunoassays [51,92,93]. Zhao and co-workers designed a transparent, lateral-flow test strip using a smartphone-based ambient-light sensor [51]. In this study, butyrylcholinesterase (BChE) was used as a model enzyme and ethyl paraoxon was a kind of typical organophosphorus (OP) pesticides. Later, the concentration of organophosphorus pesticides was detected by simultaneously measuring the total amount and activity of BChE. PtPd nanoparticles were applied in the measurement of the total amount of BChE, which could oxidize catechol to form dark brown products (Figure 2B). 

##### Competitive Method

For the detection of actual samples, the detection method is required to have good reproducibility, accuracy, and practicability. Liu and co-workers synthesized the magnetic Prussian blue nanozyme (MPBN) to apply in a bifunctional label that mimics peroxides, thereby establishing a dual-read on-demand multiplex lateral-flow immunoassay (MLFIA) to monitor β-adrenoceptor agonists [52]. In addition, other advanced technologies have been introduced in the LFIA. Ruan and co-workers utilized 3D-printing technology to design a multiplex immunosensor for the simultaneous detection of atrazine and acetochlor [53]. The quantitative detection of herbicide residues was achieved by competitive immunochromatography (ICA)-based nanozymes (mesoporous core-shell palladium@platium nanoparticles). Since the catalytic activity of Pd@Pt NPs for the reduction reaction between thiamine acetate and hydrogen peroxide provided an electrochemical driving signal, the level of herbicide residues could be further accurately detected by analyzing the electrochemical signal (Figure 2C). The combination of LFIA technology with an electrochemical analyzer with 3D-printing technology offered a rapid, precise, economical, and portable detection device for herbicide detection, which had promising applications in chemical analysis, biosensors, and point-of-care monitoring. In ICA, some nanomaterials with natural enzymatic activity can not only catalyze the color development of the substrate but also exhibit colors themselves [54,94]. Wei and co-workers synthesized Au@Pt nanozymes with peroxidase activity by a one-step method [54]. When used Au@Pt as a visual label, the anti-streptomycin (STR) combined with Au@Pt competed with the analyte STR for coating antigens (OVA-STR) on the T line. After adding the chromogenic substrates, Au@Pt could catalyze the chromogenic substrate to show color change, and then an enhanced lateral-flow immunoassay method (LFA) was established.

### 2.2. Fluorescent Immunoassays Based on Nanozymes

**Figure 3:**
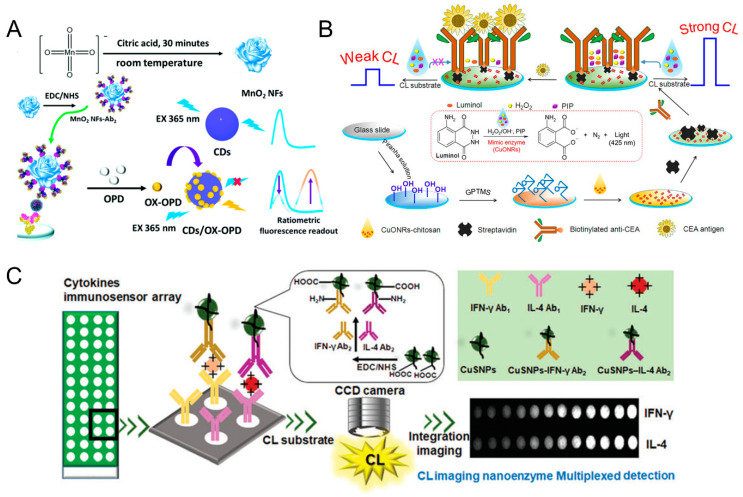
(**A**) Schematic diagram of a ratiometric fluorescence ELISA for CRP detection based on MnO2 NFs coupled with CDs [55]. Reprinted with permission from ref. [55]. Copyright 2018 Royal Society of Chemistry. (**B**) Schematic illustration of the label-free nanozyme-based chemiluminescent immunosensor by using CuONRs for CEA detection [58]. Reprinted with permission from ref. [58]. Copyright 2018 Elsevier. (**C**) Schematic illustration of CL imaging nanozyme immunoassay of multiple chicken cytokines and an immunosensor array [60]. Reprinted with permission from ref. [60]. Copyright 2018 Royal Society of Chemistry.

Fluorescence immunosensor employs an immunoreagent as a molecular recognition unit and fluorescent reagent or enzyme as a marker to measure antigens or antibodies through a specific reaction between antibody and antigen. Compared with colorimetric immunoassays, fluorescent immunoassays provide higher-sensitivity performance. Instead of natural enzyme, nanozymes (such as mimic peroxidase or oxidase) can be utilized to catalyze the oxidization of the non-fluorescent substrates to products with strong fluorescence, which can be incorporated in the fluorescent immunoassays [55,56]. However, a single fluorescence signal is easily interfered with by exterior conditions. The ratiometric fluorescence strategy is an alternative that provide built-in self-calibration via combining two emissions (a reference and a detection signal) to eliminate the interferences from the instruments and environments [95]. For instance, a ratiometric fluorescence nanozyme-based immunosorbent assay was constructed for C-reactive protein (CRP) detection, using manganese dioxide nanoflowers (MnO_2_ NFs) with oxidase-like activity to couple exogenous carbon dots (CDs) (Figure 3A) [55]. In this way, other nanozymes, such as Pd-Ir nanocubes’, with peroxidase-like activity were also employed to design a ratiometric fluorescence immunosensor with the assistance of CDs [56]. To further enhance the sensitivity of the fluorescent immunoassays, an amplification strategy, such as bio-barcode technology, can be introduced. Chen and co-workers [57] designed a competitive bio-barcode immunoassays based on a bimetallic Au@Pt nanozyme to detect organophosphate pesticides (OPs), which utilized AuNP signal amplification technology and Au@Pt catalysis. The released Au@Pt probe could directly catalyze the non-fluorescent substrate of Amplex™ Red (AR) into fluorescent resorufin for OPs without a classic enzyme labelling procedure.

### 2.3. Chemiluminescence Immunoassays Based on Nanozymes

Chemiluminescence (CL) is an emission that is generated from chemical reactions without light excitation, and detection based on the CL signal shows the advantages of high sensitivity, fast response and wide dynamic range [96]. By integrating CL signal with an immunoassay, chemiluminescence immunoassays (CLIA) can enhance the detection sensitivity by two to three orders of magnitude due to their catalytic amplification, along with high selectivity due to the specific binding of antigen–antibody [97]. The traditional CLIA analysis usually requires the enzyme to be labeled on the antigen or antibody. For instance, horseradish peroxidase (HRP) is used as an enzyme label, which catalyzes the luminol–H_2_O_2_ system to achieve a CL signal [60]. However, in the natural enzymes exist disadvantages such as poor stability, high cost, and easy denaturation. Therefore, the development of nanozymes (peroxidase mimics) to replace the natural enzyme is an alternative in CLIA. To avoid the sophisticated label method, Li and co-workers reported an efficient label-free chemiluminescent immunosensor by employing dual-function cupric oxide nanorods (CuONRs) synthesized by a simple hydrothermal method, which served as not only a mimetic peroxidase to catalyze the CL reaction but also as a solid support for the immobilization of biomolecules and recognition elements (Figure 3B) [58]. First, CuONRs were deposited onto epoxy-modified glass slides, followed by immobilizing biotinylated capture antibodies through a streptavidin bridge. Then large immunocomplexes were formed upon introducing antigens into the system, which could prevent CL substrate access to the surface, thereby reducing the CL signal. Using the carcinoembryonic antigen (CEA) as a model analyte, the proposed label-free immunosensor was able to rapidly determine CEA with a wide linear range of 0.1 to 60 ng mL^−1^, with a low detection limit of 0.05 ng mL^−1^.

To achieve highly sensitive and portable chemiluminescence detection, the CLIA can be integrated with lateral flow assays. For instance, Liu and co-workers fabricated a novel nanozyme-based chemiluminescence paper assay to develop a high-sensitive point-of-care testing (POCT) method for SARS-CoV-2 antigen detection [59]. The as-prepared Co-Fe@hemin-peroxidase could catalyze the luminol–H_2_O_2_ system to generate chemiluminescence, which was comparable to natural HRP, amplifying the immune reaction signal. To realize simultaneous multiplex detection, the CLIA could also combine with a cooled low-light charge-coupled device (CCD) to develop spatially resolved sensing arrays. Based on this, chemiluminescence imaging nanozyme immunoassay (CINIA) was proposed, wherein the nanozymes (carboxylated copper monosulfide nanoparticles, CuSNPs) were exploited as catalytic tags for monitoring multiplexed cytokine [60]. By simply conjugating CuSNPs with specific secondary antibodies, two nanozyme probes were achieved, for the sensitive detection of two chicken cytokines (IFN-γ and IL-4) in serum samples. This method avoids the complex native enzyme labelling process and achieves a high-throughput and sensitive multiplex immunoassay (Figure 3C). 

Different from the above peroxidase-like nanozyme-based immunoassays, some alkaline phosphatase-like nanozyme-based immunoassays have been developed for chemiluminescent detection. Huang and co-workers used nanoceria as the alkaline phosphatase-like catalytic label and CDP-star (phenol,2-chloro-5-(5′-chloro-4-methoxyspiro [1,2-dioxetane-3,2′-tricyclo [3.3.1.13,7]decan]-4-yl)-,1-(dihydrogen phosphate), sodium salt (1:2)) as the substrate [61]. In this case, the chemiluminescent detection of prostate specific antigen was demonstrated. What is more, the addition of ionic liquids could improve the thermal stability of enzymatic and thus increase the sensitivity of the immunoassay with a limit of detection of 53 fg mL^−1^. 

### 2.4. Electrochemical Immunoassays Based on Nanozymes

**Figure 4:**
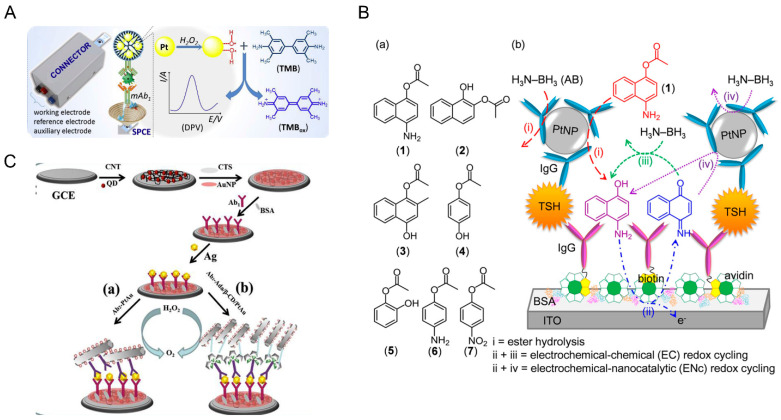
(**A**) Schematic diagram of the construction of electrochemical immunosensing platform by using PtDEN for lung cancer biomarker detection [62]. Reprinted with permission from ref. [62]. Copyright 2020 Elsevier. (**B**) Schematic of a sandwich-type electrochemical immunosensor for thyroid-stimulating hormone detection using the catalytic ester hydrolysis function of PtNP and AB [64]. Reprinted with permission from ref. [64]. Copyright 2020 Wiley Online Library. (**C**) Schematic of a near-infrared ECL immunoassay for detection of PRRSV [67]. Reprinted with permission from ref. [67]. Copyright 2017 Elsevier.

Electrochemical immunoassays have been designed based on the interaction between antigen and antibody to change the electrochemical signal, such as current, resistance, or potential response [98]. Due to the merits of low cost, high sensitivity, excellent selectivity, and good portability, electrochemical immunoassays have attracted great attention in the immunosensing field [23]. However, the electrochemical signal obtained from the interaction between antigen and antibody is relatively low; thus, the development of signal amplification strategies is urgent. As a typical signal amplifier, natural enzymes have been widely used in immunoassays, while they are unstable and easily deactivated. The nanozyme is an alternative, which can catalyze the redox of substrates like H_2_O_2_, generating highly sensitive and stable electrochemical signals for the quantitative determination of various targets. The sandwich-type assay is a common detection mode in electrochemical immunosensors [99]. Zhi et al. designed a novel electrochemical immunosensing platform, using platinum nanoparticles encapsulated inside dendrimers (PtDEN) as a peroxidase-like nanozyme for signal amplification to monitor lung cancer biomarkers (pro-gastrin-releasing peptide; ProGRP) sensitively (Figure 4A) [62]. Regarding the formation of the immunocomplex, the anti-ProGRP secondary-antibody-labeled PtDENs could oxidize TMB in the presence of H_2_O_2_ to generate a voltammetric electrochemical signal. Compared with platinum nanoparticles alone, PtDEN could improve analytical performance due to the high-efficiency catalytic efficiency from platinum nanoparticles and high-loading ability from the dendrimer. To further enhance the detection sensitivity, a dual-amplified electrochemical immunosensor has been constructed based on hemin/G-quadruplex interlaced onto Fe_3_O_4_-AuNPs or hemin-amino-reduced graphene oxide nanocomposites (H-amino-rGO-Au) [98]. In addition, competitive electrochemical immunosensors have also been developed [100]. For instance, a novel signal amplification strategy was proposed based on the competitive reaction between 2D Cu-TCPP(Fe) and polyethyleneimine (PEI) for the detection of sulfonamide [63]. Different from other electrochemical immunosensors, the 2D Cu-TCPP(Fe) nanozyme with peroxidase-like property was directly modified on the electrode surface, of which structure would be destroyed by PEI from PEI-GO@Ab2, resulting in the inhibition of catalytic activity along with the decreased electrochemical current. 

Up to now, most of the nanozyme-based electrochemical immunoassays employ peroxidase-like nanozymes for signal amplification. Recently, Nandhakumar and co-workers proposed an esterase-like nanozyme system based on Pt nanoparticles (PtNP), H_3_N-BH_3_ (AB), 4-aminonaphthalene-1-yl, to catalyze non-redox ester hydrolysis in the presence of AB (Figure 4B) [64]. Compared with natural enzymes, this nanozyme system provided higher electrochemical signal-to-background, which could be applied in the sensitive electrochemical immunosensor for thyroid-stimulating hormone (TSH) detection.

### 2.5. Electrochemiluminescence Immunoassays Based on Nanozymes

Electrochemiluminescence (ECL) immunoassay that combines ECL analysis with immune reaction has both high sensitivity of luminescence analysis and high specificity to antigen–antibody reaction. Due to the high catalytic stability, nanozymes are considered in the construction of high-performance ECL immunoassays. For instance, an enzyme-free ECL immunosensor was developed using chitosan to immobilize lead sulfide nanocrystals (PbS NCs) and combine anti-alpha fetoprotein (AFP) for the detection of AFP. The lead sulfide nanocrystals (PbS NCs) nanozymes could be used to electrochemically catalyze the luminol–H_2_O_2_ system, which could also enhance the ECL response of luminol [65]. To further improve the detection sensitivity, a dual-amplified ECL immunosensor based on PtCo nanozymes/CdS nanocrystals@graphene oxide (PtCo/CdS@GO) luminophores and K_2_S_2_O_8_/H_2_O_2_ coreactants has been designed for the ultrasensitive detection of anti-myeloperoxidase antibody (anti-MPO) [66]. Further, to avoid being interfered with by background signals of biosamples to the traditional ECL emitters, near-infrared (NIR, emission 650–900 nm) ECL immunoassays are exploited. Shao et al. prepared a porous Au/PtAu bimetallic heterojunction nanotube (PtAu BNT), which was integrated with the near-infrared ECL system (core-shell CdTe/CdS QDs-H_2_O_2_) to detect porcine reproductive and respiratory syndrome virus (PRRSV) (Figure 4C). The detection sensitivity could be enhanced significantly. On one hand, the porous PtAu BNTs served as mimic peroxidase to catalyze the NIR ECL immunoassay. On the other hand, a stable “bridge” was formed to assemble more PtAu BNTs onto the PRRSV antibody, contributing to the signal amplification [67]. 

### 2.6. SERS Immunoassays Based on Nanozymes

As a kind of optical sensing technology related to the rough surface of nanomaterials, surface-enhanced Raman spectroscopy (SERS) has the advantages of high sensitivity and in situ non-invasion and possesses a unique molecular fingerprint spectrum, which has been widely used in immunoassays. Considering the enzyme-mimicking activity of nanozymes in signal generation/amplification, nanozymes have been used as antibody labels in SERS-based immunoassays with improved detection performance. Sian and co-workers constructed a nanozyme-based SERS immunosensor for the detection of human C-reactive protein (CRP), using silver nanoparticles (Ag-NPs) as peroxidase enzymes to supersede HRP in ELISA (named SLISA) [68]. Ag NPs were conjugated to antibodies that recognized the corresponding target antigen molecule CRP and used to catalyze the oxidation of TMB by H_2_O_2_. The colored oxidation products obtained were detected by SERS.

**Figure 5:**
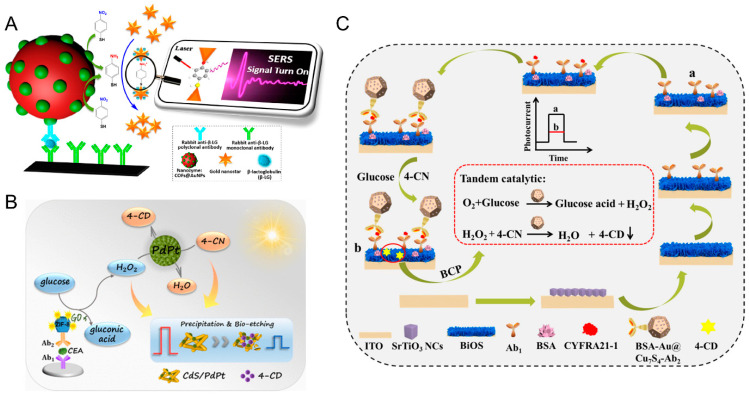
(**A**) Schematic illustration of nanozyme-based ratiometric SERS immunosorbent assay by using AuNPs doped COFs for detection of food allergy proteins [70]. Reprinted with permission from ref. [70]. Copyright 2019 American Chemical Society. (**B**) Schematic illustration of the CdS/PdPt based PEC immunoassay for CEA detection [72]. Reprinted with permission from ref. [72]. Copyright 2021 American Chemical Society. (**C**) Schematic illustration of the construction of sandwichtype ultrasensitive PEC immunosensor using a nanozyme with duple enzyme activity [73]. BiOS: Bi10O6S9 nanosheet arrays; 4-CN: 4-chloro-1-naphthol; 4-CD: enzo-4-chlorohexadienone; CYFRA: cytokeratin 19 fragment 21-1. Reprinted with permission from ref. [73]. Copyright 2021 Elsevier.

In order to realize better SERS signal amplification with higher detection sensitivity and accuracy and less background interference, researchers tried to obtain Raman active substances [e.g., malachite green (MG), 4-aminothiophenol(4-ATP)] catalyzed by nanozymes. Then Raman active substances react with gold nanoparticles to generate Raman “hot spots”, achieving the secondary amplification of analyte signals. Zhang and co-workers synthesized a peroxidase mimicking CeO_2_ nanozyme to catalyze the oxidation of a Raman inactive reporter [i.e., leucomalachite green (LMG)] in the presence of H_2_O_2_ to produce the Raman-active substance malachite green (MG) [69]. Then the Raman active substance MG reacted with gold nanoparticles to generate Raman “hot spots”, which could enhance the Raman signal for the quantification of food-allergy proteins. To further reduce the interference and enhance the detection sensitivity, a ratiometric SERS-based immunosorbent assay of allergenic protein was developed based on the gold-nanoparticles-doped covalent organic frameworks (COFs) with excellent mimic nitroreductase activity (Figure 5A). 4-nitrothiophene (4-NTP) was introduced as the substrate, which can be converted into 4-ATP in the presence of NaBH_4_ [70]. In this SERS assay, 4-ATP served as a strong bridge that could connect the gold nanoparticles through the Au–S bond and electrostatic force to further generate Raman “hot spots”. At the same time, the Raman signal of 4-nitrothiophenol at 1573 cm^−1^ was weakened, and a new signal at 1591 cm^−1^ generated by 4-ATP was turned on, leading to the generation of a ratiometric SERS signal.

### 2.7. Photoelectrochemical Immunoassays Based on Nanozymes

Photoelectrochemistry refers to the conversion of light energy into electricity by molecules, ions, or semiconductor materials that absorb photons and excite electrons to produce charge transfer. Photoelectrochemical (PEC) immunoassay is essentially the same as electrochemiluminescence (ECL) immunoassay, but the detection process is opposite. The PEC immunoassay uses the optical signal as the excitation source and detects the electrochemical signal. This different energy forms of the input and output signals renders the PEC immunoassay with a low background signal and can achieve quite high sensitivity [101]. In addition, the PEC immunoassay just needs a a simple device with the merits of simple operation, low cost, and easy micromation, which is well suited for high-throughput and real-time biological analysis. In order to gain high sensitivity, several signal amplifiers, such as natural enzymes [HRP, glucose oxidase (GOx), or alkaline phosphatase (ALP)], have been used in the labeling of PEC immunoassays, achieving enzyme-mediated signal amplification. In recent years, nanozymes have become a good substitute for natural enzymes because of their low cost and high stability, serving as labels conjugated with signal antibodies to significantly amplify the photocurrent signal. Using a high-activity Fe_3_O_4_ nanozyme as the signal amplifier and ZnIn_2_S_4_/ZnO-NRs/ITO photoelectrodes as the PEC matrix, a simple, low-cost PEC immunoassay for the ultrasensitive detection of target biomarkers was developed [71]. In addition, through the efficient enzymatic reactions and noticeable enhancement in the photoresponse, highly active nanozymes based on noble metals could greatly amplify PEC biosensing signals. Chen and coworkers proposed a sensitive split-type glucose oxidase-mediated PEC immunoassay for carcinoembryonic antigen (CEA) detection, using PdPt bimetallic nanozymes coupled with CdS photoactive material (Figure 5B) [72]. Since the visible-light absorption capability of the PdPt system is superior to the pristine CdS, the system possesses enhanced PEC performance. Specifically, the GOx and secondary antibodies (Ab2) were conjugated with zeolitic imidazolate framework-8 (ZIF-8). Upon the addition of CEA, a sandwich immunocomplexing was formed, to induce the generation of H_2_O_2_ by the confined GOx labels. Then the outstanding peroxidase-like properties of PdPt bimetallic nanozymes enabled the catalyzed oxidation of 4-chloro-1-naphthol (4-CN) to generate insoluble precipitates and initiated the enzymatic bioetching of CdS nanorods in the presence of H_2_O_2_, leading to the suppressed photocurrent signalin synergistic ways. However, the development of ultrasensitive PEC immunoassays requires a signal regulation strategy, and biocatalytic precipitation (BCP) is as a convenient and effective method, which uses enzymatic reaction to form an insoluble precipitate to influence the PEC reaction. To simplify the detection system, a sandwich-type ultrasensitive PEC immunosensor relying on a multiple signal inhibition strategy was developed, using a nanozyme with duple enzyme activity (Figure 5C) [73]. There is an effective PEC signal regulator that the bovine serum albumin stabilized gold nanoparticles (BSA@Au NPs) anchored on Cu_7_S_4_ polyhedron composite (BSA-Au@Cu7S4 polyhedron). Furthermore, BSA@Au NPs is a duple enzyme that mimics the activity of glucose oxidase (GOx) and peroxidase, inducing interesting tandem BCP to reduce the PEC signal. Moreover, the competitive light absorption between the BSA-Au@Cu7S4 polyhedron and photoactive matrix caused a further reduction in the PEC signal. Consequently, a variety of signal suppression strategies such as tandem BCP, competitive light absorption, and steric hindrance are usually used to quench the PEC signal quickly, which improves the detection sensitivity of the PEC immunosensor. This technique has unlimited potential in the analysis of biological samples. 

### 2.8. Other Immunoassays Based on Nanozymes

**Figure 6:**
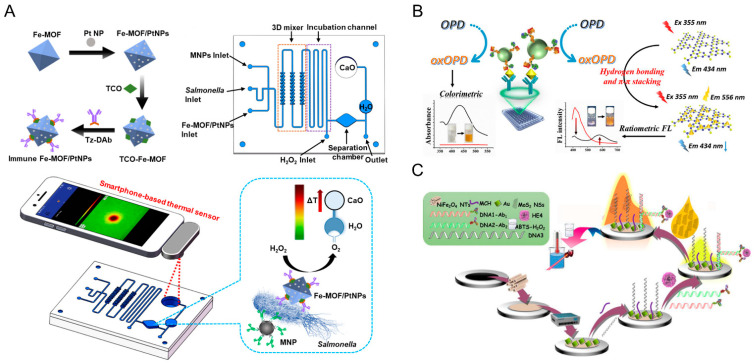
(**A**) Schematic diagram of the microfluidic immunosensor for *Salmonella* detection [75]. Reprinted with permission from ref. [75]. Copyright 2021 American Chemical Society. (**B**) Schematic illustration of the principle of nanozyme-linked immunosorbent assay for dual colorimetric and ratiometric fluorescent determination of cTnI [76]. Reprinted with permission from ref. [76]. Copyright 2019 Elsevier. (**C**) Schematic illustration of the proximity hybridization-based multiple stimuli-responsive immunosensing platform for ECL, colorimetry and photothermal sensing of ovarian cancer biomarkers [78]. Reprinted with permission from ref. [78]. Copyright 2020 Elsevier.

In the past, the traditionally used signals in immunosensor research include colorimetry, fluorescence, electrochemistry, etc. Although these methods can provide sensitive detection results, most of them require expensive instruments, professional operators, and a high operating level. For the detection of various target analytes, real-time, rapid, and sensitive detection methods with novel detection signals are urgently needed in practical applications. In order to obtain high sensitivity, nanozymes can be applied to a signal probe to amplify the signal. Inspired by the “elephant toothpaste” experiment, Liu and co-workers developed a novel and low-cost portable foam immunoassay method [74]. The peroxidase-like nanozymes’ gold and platinum nanoparticles (Au@Pt NP) catalyzed the foam generation reaction to produce a sensitive height readout. The sensitivity of *Escherichia coli* (*E. coli* O157:H7) could be measured by a linear relationship between the height of the foam and the logarithm of the *E. coli* O157:H7 concentration. In addition, based on the thermal sensor of a smartphone, a microfluidic immunosensor was designed for *Salmonella* with a double-layer microfluidic chip as the research platform and Fe-MIL-88NH_2_ metal−organic framework nanocubes decorated with platinum nanoparticles (Fe-MOF/PtNPs) as the signal amplifier (Figure 6A) [75]. Herein, the conjugate formation of an immune magnetic nanoparticles (MNPs)–bacteria–Fe–MOF/PtNPs sandwich complex in a microfluidic chip could catalyze H_2_O_2_ to generate O_2_, to increase the pressure and promote the reaction between preloaded water and calcium oxide (CaO) powder to produce heat. Finally, the thermal sensor of a smartphone could be used to determine bacteria, and the detection limit is 93 CFU/mL.

### 2.9. Multimodal Immunoassays Based on Nanozymes

A multimodal immunosensor is an analysis method based on the conjugation of the immune-recognition system with a multimodal detection signal. The sensor in the process of establishing combines several signal probes that make it output multiple sensing signals under the same or different analysis conditions. It not only has the unique advantages of a multimodal sensor with high accuracy, large information flux, and small sample consumption but also features an immunoassay with high sensitivity and good specificity. Nanozymes can participate in catalyzing some reactions and provide colorimetric, fluorescent, photothermal, and electrochemical response, etc. [76,77,78,102,103,104]. Therefore, the combination of dual mode (such as colorimetric/fluorescent, colorimetric/electrochemical, colorimetric/photothermal, colorimetric/SERS) or even muti-mode (colorimetric/photothermal/chemiluminescent) immunoassays have emerged recently. Miao and co-workers designed a robust nanozyme-linked immunosorbent assay with ratiometric fluorescent and colorimetric signal output that can achieve the sensitive, accurate, and reliable detection of human cardiac troponin I (Figure 6B) [76]. The nanoceria with peroxidase-like properties oxidized o-phenylenediamine (OPD) and converted it to 2,3-diaminophenazine (oxOPD). This process produced a noticeable color change that could be recorded by colorimetry. At the same time, oxOPD could effectively quench the fluorescence of graphite carbon nitride quantum dots (g-C_3_N_4_ QDs) and obtain a reliable ratiometric fluorescence readout, so the method had good self-calibration ability. Additionally, Choi and co-workers synthesized urchin-like Pt nanozymes and developed a novel nanozyme-based colorimetric–electrochemical immunoassay method for detecting glycated albumin (GA), known to reflect short-term blood sugar levels [77]. Moreover, false-positive or false-negative results can be effectively avoided by the mutual calibration of multiple signals in immunoassays. In addition, catalase-mimicking nanozymes can also be used to design a multimodal immunoassay. For example, pH-switchable nanozyme polyethyleneimine-coated Prussian blue nanocubes (PBNCs@PEI) were synthesized; they exhibited peroxidase-like activity in acidic pH and catalase-like activity in alkaline conditions [105]. The peroxidase-like activity of PBNCs@PEI in acidic pH could induce the colorimetric reaction of Au nanostars with TMB^2+^/CTAB, whereas the catalase-like activity of PBNCs@PEI in alkaline pH could catalyze the decomposition of H_2_O_2_ to produce O_2_, which could further oxidize 4-chloro-1-naphthol (4-CN) with decreased fluorescence intensity. Thus, a dual-mode (colorimetric/fluorescent) immunosensor for rosiglitazone detection was constructed based on the competitive immunoassay. With the above considerations, Zhang and co-workers creatively introduced versatile MoS_2_ nanosheets (MoS_2_ NSs), which had good electrocatalytic activity for dissolved O_2_ and good photothermal effect for increasing electrode temperature [78]. It also could be used as peroxidase to oxidate 2,2′-azinobis (3-ethylbenzothiazoline)-6-sulfonic acid (ABTS) to produce a colorimetric signal and improve the electrochemiluminescence performance of a luminol cathode. Based on this, an attractive multi-element response platform for ECL, colorimetry, and photothermal sensing was designed for the detection of ovarian cancer biomarkers in human serum. Triggering or improving multiple signals by using the same materials will become a new trend in research (Figure 6C). 

## 3. Conclusions and Perspective

In conclusion, we have summarized the current development of immunoassays with various signaling modes, wherein nanozymes are employed as catalytic labels. On one hand, the specific-recognition effect between antigens and antibodies makes the immunoassays present the feature of high detection selectivity. On the other hand, by using the nanozymes with tunable and high catalytic activities instead of natural enzymes, the detection sensitivity has been improved through various signal amplification routes. Additionally, compared with natural enzymes, nanozymes also have superiority in easy preparation, low cost, and high stability. Therefore, nanozyme-based immunoassays have been developed gradually with rapidity and robustness and can be widely applied in biosensing fields. Furthermore, the limitations and future perspective of nanozyme-based immunoassays summarized herein will inspire researchers to continuously expand the applications of immunoassays by integrating them with new materials, biotechnology, and micromodule equipment. (1) The development of new types of composite nanozyme materials. At present, most nanozymes (such as peroxidase mimic) show catalytic activity with a high dosage, and the optimal catalytic activity are exhibited under acid conditions. Thus, it is desirable to exploit novel nanozymes with superior catalytic efficiency under mild conditions. (2) Using biotechnology. Sometimes the ultra-low content of target analytes from practical samples cannot be detected by the normal immunoassays. For instance, nanozyme-based immunoassays can be combined with DNA nanotechnology to further amplify the signal and improve the detection sensitivity. (3) Integrated with micro devices. It is time to move the traditional nanozyme-based immunoassays out of the lab. By integrating micro devices such as smart phones, micro-fluidic chips with nanozyme-based immunoassays, point-of-care testing, and on-site analysis can be realized, which can improve the detection efficiency, simplify the operation procedure, and reduce the cost. Overall, the nanozyme-based immunoassays are becoming a promising area of research and have potential prospects in a wide range of applications. 

## Figures and Tables

**Table 1:** Summarization of immunoassays with diverse signals based on nanozymes for target analytes detection.

Nanozymes	Signals	Analyte	Limit of Detection (LOD)	Comparison of Sensitivity with Natural Enzymes-Labeled Immunoassays	Ref.
Ps-Pt	Colorimetric	*S. typhi*	100 CFU mL^−1^	-	[24]
a-RuTe_2_ nanorods	Colorimetric	PSA	32.6 pg mL^−1^	The LOD was one order of magnitude lower than that of ELISA.	[25]
PBNPs	Colorimetric	HSA	1.2 ng mL^−1^	The LOD was improved by 3 orders of magnitude that of ELISA.	[26]
Fe_3_O_4_@SiO_2_-NH_2_-Au@Pd_0.30_NPs	Colorimetric	antip53aAbs	15 pg mL^−1^	The LOD was lower than that of ELISA (0.19 ng mL^−1^).	[27]
aptamer-AuNPs	Colorimetric	CRP	8 pg mL^−1^	The LOD was lower than that of ELISA (0.14 μg mL^−1^).	[28]
LMs	Colorimetric	α-LA	0.056 ng mL^−1^	-	[29]
TiO_2_-CA NPs	Colorimetric	ALP	0.002 U L^−1^	-	[30]
MnO_2_ nanoflakes	Colorimetric	AFP	0.148 ng mL^−1^	-	[31]
2D GO/Fe-MOF	Colorimetric	BPDE-DNA	0.268 ng mL^−1^	-	[32]
Cu(OH)_2_-DNAzymes	Colorimetric	MC-LR	6 ng L^−1^	The LOD was lower than that of ELISA (190 ng L^−1^).	[33]
Fe-N-C single-atom	Colorimetric	CEA	0.55 pg mL^−1^	The LOD was lower than that of ELISA (2 pg mL^−1^).	[34]
FeSA−PtC nanozymes	Colorimetric	PSA	1.8 pg mL^−1^	-	[35]
AuNZs	Colorimetric	influenza virus A (H1N1)	44.2 × 10^−15^ g mL^−1^	-	[36]
TMB NPs	Colorimetric	interleukin-6	0.66 pg mL^−1^	The sensitivity was 11.82 times higher than that of ELISA (7.8 pg mL^−1^).	[37]
TMBZ-AuNPs	Colorimetric	influenza virus A (H5N1)	1.11 pg mL^−1^	The LOD was lower than that of ELISA (909 pg mL^−1^).	[38]
PDA-Fe(III) NPs	Colorimetric	PSA	0.84 pg mL^−1^	The LOD was lower than that of ELISA (6.81 pg mL^−1^).	[39]
AuNSs@CTAB	Colorimetric	HCG	7.8 mIU mL^−1^	-	[40]
Au@Pt probes	Colorimetric	parathion	2.13 ng·kg^−1^	The LOD was lower than that of ELISA (20.82 ng·kg^−1^).	[41]
PVP-PtNC nanozyme	Colorimetric	AMD	0.134 ng mL^−1^	The LOD was lower than that of HRP-labeled IgG catalyzed colorimetric reactions (0.343 ng mL^−1^).	[42]
Co(OH)_2_ nanocages	Colorimetric	ochratoxin A	0.26 ng L^−1^	-	[43]
SAN	Colorimetric	cTnI-TnC Myo	0.012 ng mL^−1^ 0.2 ng mL^−1^	The LODs were lower than ELISA kit (0.03 ng mL^−1^ for cTnI, and 5 ng mL^−1^ for Myo).	[44]
Fe_3_O_4_@PDA@Pd/Pt	Colorimetric	*E. coli* O157:H7	9 × 10^1^ CFU mL^−1^	-	[45]
man-PB	Colorimetric	*E. coli* O157:H7	102 CFU mL^−1^	-	[46]
Pd@Pt	Colorimetric	*S. Enteritidis* *E. coli* O157:H7	∼20 CFU mL^−1^ ∼34 CFU mL^−1^	The LOD was 100-fold more sensitive than AuNP-based LFIAs and similar to HRP enhanced LFIAs.	[47]
PtGNs	Colorimetric	*E. coli* O157:H7	3.3 × 10^4^ CFU mL^−1^	30-fold improvement in LOD was achieved over the conventional LFA.	[48]
in situ gold growth of AuNPs	Colorimetric	*E. coli* O157:H7	1.25 × 10 CFU mL^−1^	The LOD was 400-fold lower than colloidal gold immunochromatography assay.	[49]
MNP	Colorimetric	*ES*	2 CFU mL^−1^	-	[50]
PtPd nanoparticles	Colorimetric	BChE	0.025 nM	-	[51]
MPBN	Colorimetric	CLE RAC	0.20 ng mL^−1^ 0.12 ng mL^−1^	-	[52]
Pd@Pt NP	Colorimetric	Atrazine acetochlor	0.24 ppb 3.2 ppb	-	[53]
Au@Pt nanozyme	Colorimetric	STR	1 ng mL^−1^	The LOD was lower than LFA based on AuNPs (8 ng mL^−1^).	[54]
MnO_2_ NFs	Fluorescence	CRP	0.67 pg mL^−1^	The LOD was lower than electrochemical ELISA (12.5 pg mL^−1^).	[55]
Pd-Ir nanocubes	Fluorescence	cardiac troponin I	0.31 pg mL^−1^	-	[56]
Au@Pt nanozyme	Fluorescence	parathion Triazophos chlorpyrifos	9.88 ng kg^−1^ 3.91 ng kg^−1^ 1.47 ng kg^−1^	-	[57]
CuONRs	Chemiluminescence	CEA	0.05 ng mL^−1^	The LOD was 12-fold more sensitive than HRP-labeled sandwich CLIA.	[58]
Co-Fe@hemin	Chemiluminescence	SARS-CoV-2 antigen	0.1 ng mL^−1^	-	[59]
CuSNPs	Chemiluminescence	IFN-γ IL-4	2.9 pg mL^−1^ 3.2 pg mL^−1^	The LOD was lower than ELISA (500 pg mL^−1^ for IFN-γ) and CLIA (20 pg mL^−1^ for IL-4).	[60]
nanoceria	Chemiluminescence	PSA	53 fg mL^−1^	-	[61]
PtDEN	Electrochemical	ProGRP	0.86 pg mL^−1^	The LOD was lower than EC immunosensor (10 pg mL^−1^).	[62]
2D Cu-TCPP(Fe)	Electrochemical	SAs	0.395 ng mL^−1^	-	[63]
PtNP	Electrochemical	TSH	0.3 pg mL^−1^	-	[64]
PbS NCs	Electrochemiluminescence	AFP	3.0 fg mL^−1^	The LOD was lower than ELISA (0.1 ng mL^−1^).	[65]
PtCo nanozymes	Electrochemiluminescence	anti-MPO	7.39 fg mL^−1^	-	[66]
PtAu BNT	Electrochemiluminescence	PRRSV	10.8 pg mL^−1^	-	[67]
Ag-NPs	SERS	CRP	1.09 ng mL^−1^	-	[68]
CeO_2_	SERS	α-LA	0.01 ng mL^−1^	The LOD was lower than ELISA (0.128 ng mL^−1^).	[69]
AuNPs doped COF	SERS	β-lactoglobulin	0.01 ng mL^−1^	The LOD was lower than sandwich ELISA (33.95 ng mL^−1^).	[70]
Fe_3_O_4_	Photoelectrochemical	PSA	18 fg mL^−1^	The LOD was lower than sandwich HRP labeled PEC immunoassay (37 fg mL^−1^).	[71]
PdPt bimetallic nanozymes	Photoelectrochemical	CEA	0.21 pg mL^−1^	-	[72]
BSA@Au NPs	Photoelectrochemical	CYFRA21-1	1.12 fg mL^−1^	-	[73]
Au@Pt NP	Height of foam	*E. coli* O157:H7	2.16 × 10^2^ CFU mL^−1^	-	[74]
Fe-MOF/PtNPs	Temperature	*Salmonella*	93 CFU mL^−1^	The LOD was lower than sandwich ELISA (10^4^ CFU mL^−1^).	[75]
nanoceria	Colorimetric Ratiometric fluorescence	cTnI	0.227 pg mL^−1^ 0.413 pg mL^−1^	-	[76]
Pt nanozymes	Colorimetric Electrochemical	GA	9.2 μg mL^−1^ 3.8 μg mL^−1^	-	[77]
MoS_2_ NSs	Colorimetric Electrochemiluminescence Photothermal	HE4	3 × 10^−5^ ng mL^−1^ 3 × 10^−7^ ng mL^−1^ -	-	[78]

- not mentioned. **Abbreviations:** α-LA: alpha-lactalbumin; AFP: alpha fetoprotein; Ag-NPs: silver nanoparticles; AMD: amantadine; anti-MPO: Anti-myeloperoxidase antibody; antip53aAbs: tumor-associated anti-p53 autoantibodies; aptamer-AuNPs: aptamer-functionalized gold nanoparticles; a-RuTe_2_: amorphous RuTe_2_; AuNPs doped COF: gold nanoparticles doped covalent organic frameworks; AuNZs: gold nanozyme; AuNPs@CTAB: cetyltrimethylammonium bromide (CTAB)-coated gold nanoparticles; Au@Pt NP: gold and platinum nanoparticles; BChE: butyrylcholinesterase; BPDE−DNA: benzo[a]pyrene-7,8-diol 9,10-epoxide−DNA adduct; BSA@Au NPs: bovine serum albumin stabilized gold nanoparticles; CEA: carcinoembryonic antigen; CLE: clenbuterol; Co(OH)_2_ nanocages: cobalt hydroxide nanocages; CRP: C-reactive protein; cTnI: cardiac troponin I; cTnI-TnC: I-troponin C; CuONRs: cupric oxide nanorods; CuSNPs: carboxylated copper monosulfide nanoparticles; CYFRA21-1: cytokeratin 19 fragment 21-1; 2D GO/Fe-MOF: Graphene oxide (GO)/Fe-based metal−organic frameworks (Fe-MOF); *E. coli* O157:H7: *Escherichia coli* O157:H7; *ES*: *Enterobacter sakazakii;* FeSA-PtC nanozyme: Pt clusters loading on the Fe single-atom; Fe_3_O_4_@SiO_2_-NH_2_-Au@Pd_0.30_NPs: nanomagnet-silica nanoparticles decorated with Au@Pd; Fe_3_O_4_@PDA@Pd/Pt: polydopamine (PDA)-mediated magnetic bimetallic nanozyme; Fe-MOF/PtNPs: Fe-MIL-88NH_2_ metal−organic framework nanocubes decorated with platinum nanoparticles; GA: glycated albumin; HCG: human chorionic gonadotropin; HE4: human epididymis-specific protein 4; HSA: human serum albumin; LMs: laccase mimics nanozymes; man-PB: mannose modified Prussian blue; MnO_2_ NFs: manganese dioxide nanoflowers; MNP: magnetic nanoparticle; MoS_2_ NSs: MoS_2_ nanosheets; Myo: myoglobin; PbS NCs: lead sulfide nanocrystals; PtDEN: platinum nanoparticles encapsulated inside dendrimers; ProGRP: pro-gastrin-releasing peptide; PtNP: Pt nanoparticle; PtAu BNT: Au/PtAu bimetallic heterojunction nanotube; PRRSV: porcine reproductive and respiratory syndrome virus; MC-LR: microcystin-LR; MPBN: magnetic prussian blue nanozyme; PBNPs: Prussian blue nanoparticles; PDA: polydopamine (PDA) nanoparticle; Pd@Pt NP: palladium@platium nanoparticle; Ps-Pt: platinum-nanoparticles on the surface of carboxyl functionalized polymer nanospheres; PSA: prostate-specific antigen; PtGNs: Platinum-coated gold nanozymes; PVP-PtNC nanozyme: polyvinylpyrrolidone (PVP) capped Pt nanocubes; RAC: ractopamine; SAs: sulfonamides; SANs: self-assembled allochroic nanoparticles; *S. typhi*: Salmonella typhimurium; STR: streptomycin; *S. Enteritidis*: *Salmonella Enteritidis*; TMB NPs: Allochroic 3,3′,5,5′-tetramethylbenzidine (TMB) nanoparticles; TMBZ-AuNPs: 3,3′,5,5′- Tetramethylbenzidine (TMBZ)-gold nanoparticles; TiO_2_-CA NPs: CA coordinated TiO_2_; TSH: thyroid-stimulating hormone.

## Data Availability

Not applicable.
